# Risk of peripheral vestibular disorders and hearing impairment among users of glucagon-like peptide-1 receptor agonists

**DOI:** 10.7150/ijms.131397

**Published:** 2026-03-17

**Authors:** Jing-Yang Huang, Yen-Ting Lu, Chung-Han Hsin, Tun-Shin Lo, Chia-Yi Lee, Shun-Fa Yang, Miao-Yu Liao

**Affiliations:** 1 Department of Nursing, Chung Shan Medical University, Taichung, Taiwan.; 2 Institute of Medicine, Chung Shan Medical University, Taichung, Taiwan.; 3 Department of Medical Research, Chung Shan Medical University Hospital, Taichung, Taiwan.; 4 School of Medicine, Chung Shan Medical University, Taichung, Taiwan.; 5 Department of Otolaryngology, Chung Shan Medical University Hospital, Taichung, Taiwan.; 6 Department of Otolaryngology, St. Martin De Porres Hospital, Chiayi, Taiwan.; 7 Department of Speech Language Pathology and Audiology, Chung Shan Medical University, Taichung, Taiwan.; 8 Department of Otolaryngology, Chung Shan Medical University Hospital, Taichung, Taiwan.; 9 Department of Family Medicine, Taichung Hospital, Ministry of Health and Welfare, Taichung, Taiwan.

**Keywords:** glucagon-like peptide-1 receptor agonists, peripheral vestibular disorder, hearing impairment, type 2 diabetes mellitus, epidemiology

## Abstract

Glucagon-like peptide-1 receptor agonists (GLP-1 RAs) are widely used antidiabetic agents with established glycemic efficacy. Peripheral vestibular disorders and hearing impairment are inner ear conditions that may be influenced by metabolic and glycemic status. This study aimed to investigate the association between GLP-1 RA use and the development of peripheral vestibular disorders, dizziness, and hearing impairment. This retrospective cohort study identified patients who received GLP-1 RA treatment and matched them with non-GLP-1 RA users. After propensity score matching, a total of 684,092 participants were included, with 342,046 in the GLP-1 RA group and 342,046 in the non-GLP-1 RA groups. The primary outcomes were newly diagnosed peripheral vestibular disorders, dizziness, and hearing impairment. Cox proportional hazards regression models were applied to compare outcome incidences between groups. During the follow-up period, 4,407 cases of peripheral vestibular disorders occurred in the GLP-1 RA group compared with 3,946 cases in the non-GLP-1 RA group, indicating a significantly higher incidence among GLP-1 RA users (P < 0.001). Dizziness was diagnosed in 32,545 GLP-1 RA users and 29,931 nonusers, while hearing impairment occurred in 9,522 and 7,898 participants, respectively; both outcomes were significantly more frequent in the GLP-1 RA group (P < 0.001). Cumulative incidence analyses showed significantly higher risks for all three outcomes in the GLP-1 RA group. In conclusion, GLP-1 RA use may presented be with higher incidences of peripheral vestibular disorders, dizziness, and hearing impairment in white population.

## Introduction

Type 2 diabetes mellitus (T2DM) is a metabolic disease that has a substantial impact on public health worldwide 1. Insulin resistance and chronic hyperglycemia are the primary characteristics of T2DM and can lead to damage in multiple organ systems 2. Patients with T2DM have a significantly increased risk of cardiovascular diseases, which may result in increased mortality 3. In addition, microvascular complications, including diabetic retinopathy, diabetic nephropathy, and diabetic neuropathy, are associated with severe tissue damage and functional impairment 3, 4. Consequently, the management of T2DM is a critical issue that cannot be overemphasized.

Several pharmacological agents are currently available for the management and control of T2DM 1, 2, 5. Metformin has been prescribed for decades and remains a first-line antidiabetic medication with well-established efficacy and safety 6. More recently, sodium-glucose cotransporter 2 inhibitor (SGLT2i) have demonstrated effective glycemic control in patients with T2DM 7. In addition, dipeptidyl peptidase-4 inhibitor (DPP-4i) are favorable antihyperglycemic agents and have shown significant reductions in blood glucose levels in previous studies 8. Glucagon-like peptide-1 receptor agonists (GLP-1 RAs) are another class of anti-diabetic medications that have gained popularity, demonstrating better outcomes compared to older anti-diabetic drugs 9, 10.

Although GLP-1 RAs have demonstrated excellent therapeutic outcomes in T2DM, including reductions in comorbidities 11, several adverse effects have been reported following their use 12. Delayed gastric emptying, resulting in gastrointestinal discomfort, is a well-recognized complication associated with GLP-1 RA therapy 12. In addition, a potential association between GLP-1 RA use and thyroid cancer has been suggested 13. Nevertheless, few studies have investigated the relationship between GLP-1 RA use and the development of inner ear disorders. Given that certain antidiabetic medications including GLP-1 RA and sodium-glucose cotransporter 2 inhibitors may exert ototoxic effects 14, such an association is biologically plausible and warrants further investigation.

Accordingly, the aim of the present study was to evaluate the potential association between GLP-1 RA use and the development of inner ear diseases, including peripheral vestibular disorders and hearing impairment. Relevant confounders associated with these conditions were also incorporated and adjusted for in multivariable analyses.

## Materials and Methods

### Ethics Declaration

All procedures in the present study were conducted in accordance with the Declaration of Helsinki of 1964 and its subsequent amendments. The study protocol was approved by the Institutional Review Board of Chung Shan Medical University Hospital (Project code: CS1-24047). The requirement for written informed consent was waived by the Institutional Review Board because all data were de-identified.

### Data Source

This retrospective cohort study was conducted using data from the TriNetX Research Network, a federated electronic health record-based research platform that includes large academic and community healthcare organizations. The TriNetX database contains comprehensive electronic medical records, including diagnoses, laboratory values, procedures, medications, and selected genomic information. The data used in this study were derived from the US Collaborative Network, which comprises approximately 110 healthcare organizations. TriNetX aggregates and de-identifies electronic health records from participating healthcare systems, most of which are large academic institutions providing both inpatient and outpatient care across diverse regions of the United States.

The TriNetX Analytics platform provides secure, web-based access to longitudinal clinical data from primary care, hospital, and specialty providers. Consequently, the database encompasses a wide range of age groups, geographic regions, racial and ethnic populations, insurance categories (including Medicare, Medicaid, military insurance, workers' compensation, self-pay, uninsured, Veterans Affairs insurance, and employer sponsored insurance), and income levels. Available data elements include International Classification of Diseases, Tenth Revision, Clinical Modification (ICD-10-CM) codes, demographic characteristics, socioeconomic and psychosocial variables, educational attainment, hospitalization duration, imaging and surgical procedure codes, laboratory test results, and medication records classified using Anatomical Therapeutic Chemical (ATC) codes.

### Participant Selection

The study population included adults aged ≥18 years with a diagnosis of T2DM identified using ICD-10-CM codes who had at least one clinical encounter between January 1, 2016, and December 31, 2024. Treatment initiation was defined as the index date. A 90-day stabilization window surrounding the index date was applied to minimize exposure misclassification due to early treatment switching or combination therapy and to ensure robust identification of incident users. Participants were assigned to the GLP-1 RA group if they received their first recorded prescription for a GLP-1 RA during the study period according to ATC codes. The non-GLP-1 RA group included participants who initiated treatment with either a DPP-4i or a SGLT2i.

**Figure 1:**
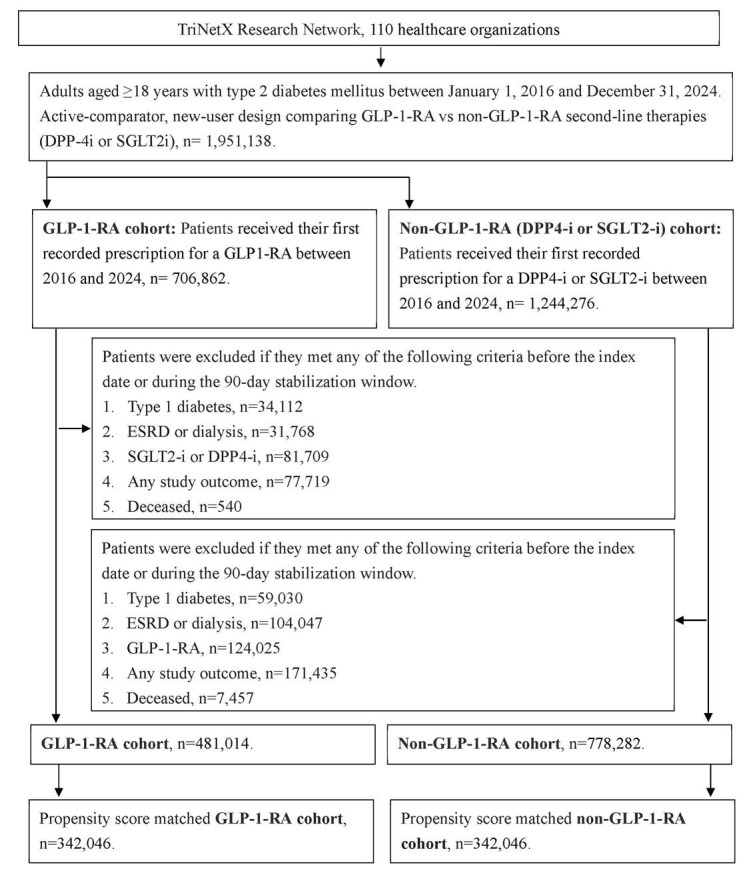
The flowchart of participant selection. N: number, T2DM: type 2 diabetes mellitus, GLP-1 RA: glucagon-like peptide 1 receptor agonist, PSM: propensity score-matching.

To enhance cohort homogeneity and reduce confounding, participants were excluded if any of the following conditions occurred before the index date or within the stabilization window: (1) diagnosis of type 1 diabetes mellitus; (2) end-stage renal disease or dialysis dependence, identified by ICD-10-CM codes, dialysis procedure codes, or an estimated glomerular filtration rate (eGFR) <15 mL/min/1.73 m²; (3) exposure to comparator treatments (i.e., DPP-4i or SGLT2i for the GLP-1 RA group); (4) occurrence of study outcomes; or (5) death according to TriNetX mortality data. Propensity score matching (PSM) was performed to balance baseline characteristics between the GLP-1 RA and non-GLP-1 RA groups using the PROC PSMATCH procedure in SAS. Propensity scores representing the probability of GLP-1 RA exposure were estimated using logistic regression incorporating multiple covariates. Each GLP-1 RA user was matched 1:1 to a nonuser using a nearest-neighbor greedy algorithm with a caliper width of 0.01. After matching, 342,046 participants were included in each group. The participant selection process is illustrated in Figure 1.

### Primary Outcome

The primary outcomes were the development of peripheral vestibular disorders, dizziness, and hearing impairment. Peripheral vestibular disorders included Ménière's disease, benign paroxysmal positional vertigo, vestibular neuronitis, and labyrinthitis, identified using corresponding ICD-10-CM codes. Dizziness was identified using relevant ICD-10-CM codes. Hearing impairment included sensorineural hearing loss, ototoxic hearing loss, and presbycusis. Of note, all the "peripheral vestibular disorders" "dizziness" and "hearing impairment" episode was diagnosed by an otolaryngology specialist. To improve diagnostic validity, only participants with repeated diagnostic records were considered to have achieved the study outcomes.

### Predisposing Factors

Baseline covariates were assessed during the 12-month period preceding the index date and included age, sex, race and ethnicity, body mass index (BMI), healthcare utilization, socioeconomic and psychosocial factors, comorbid conditions, medication use, and laboratory values. Comorbidities included hypoglycemia, dyslipidemia, hypertension, liver disease, ischemic heart disease, heart failure, cerebrovascular disease, transient ischemic attack, chronic lower respiratory disease, psychiatric disorders, rheumatoid arthritis, head injury, substance use disorders, migraine, and otitis media. Medication variables included antidiabetic agents, cardiovascular medications, ototoxic drugs, weight-loss medications, and chemotherapy agents. Surgical history included bariatric surgery and middle ear procedures. Laboratory measures included glycated hemoglobin (HbA1c), eGFR, and serum low-density lipoprotein cholesterol. Participants were followed until outcome occurrence, loss to follow-up within the TriNetX network, or December 31, 2024.

### Statistical Analysis

Statistical analyses were performed using SAS version 9.4 (SAS Institute Inc., Cary, NC, USA). A modified intention-to-treat approach was applied, with follow-up beginning on day 91 after the index date. Baseline characteristics were summarized descriptively, and standardized mean differences (SMDs) were used to assess covariate balance, with an SMD >0.1 indicating meaningful imbalance. Cox proportional hazards regression models were used to estimate adjusted hazard ratios (aHRs) and 95% confidence intervals (CIs) for study outcomes. The proportional hazards assumption was evaluated using generalized Schoenfeld residuals. Kaplan-Meier methods were applied to estimate cumulative incidence, with appropriate censoring. Prespecified subgroup analyses were conducted according to sex, race, age, BMI, glycemic control (HbA1c), and renal function (eGFR, calculated using the CKD-EPI equation). Time-to-event analyses were conducted within the TriNetX analytics platform using the R survival package (version 3.2-3). A two-sided P value <0.05 was considered statistically significant, and P values <0.001 were reported as P < 0.001.

## Results

The baseline characteristics of the GLP-1 RA and non-GLP-1 RA groups are presented in Table 1. Before propensity score matching, a total of 1,259,296 participants were included, with 481,014 in the GLP-1 RA group and 778,282 in the non-GLP-1 RA groups. After matching, 342,046 participants were included in each group. The mean age was 58.5 ± 12.0 years in the GLP-1 RA group and 58.5 ± 12.8 years in the non-GLP-1 RA group, with no significant difference between groups (standardized mean difference [SMD] = 0.0028). Demographic characteristics, including sex, race, socioeconomic and psychosocial factors, medical encounters, and healthcare utilization, were well balanced between the two groups (all SMDs < 0.1). Regarding systemic comorbidities, the distributions of all diseases included in the analysis were comparable between groups (all SMDs < 0.1). Use of SGLT2 inhibitors and DPP-4 inhibitors was more frequent in the non-GLP-1 RA group than in the GLP-1 RA group (both SMDs > 0.1), whereas the use of other medications, surgical procedures, and laboratory values did not differ significantly between groups (all SMDs < 0.1) (Table 1).

**Figure 2:**
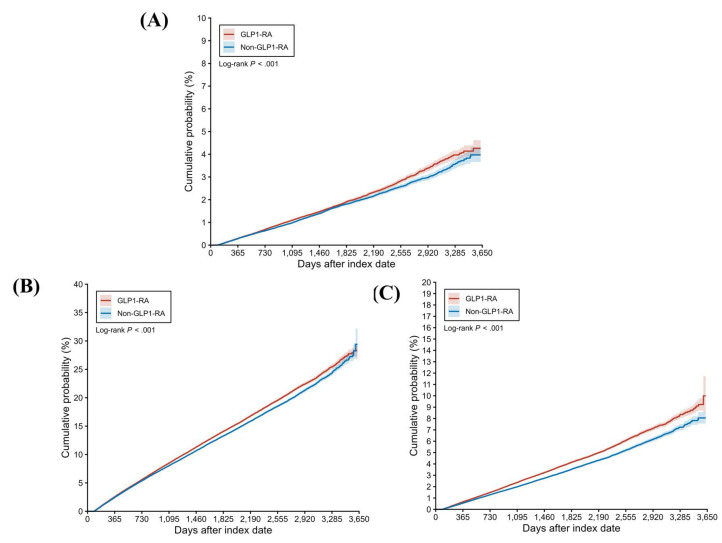
The cumulative probability of primary outcomes between the two groups. GLP-1 RA: glucagon-like peptide 1 receptor agonist.

**Figure 3:**
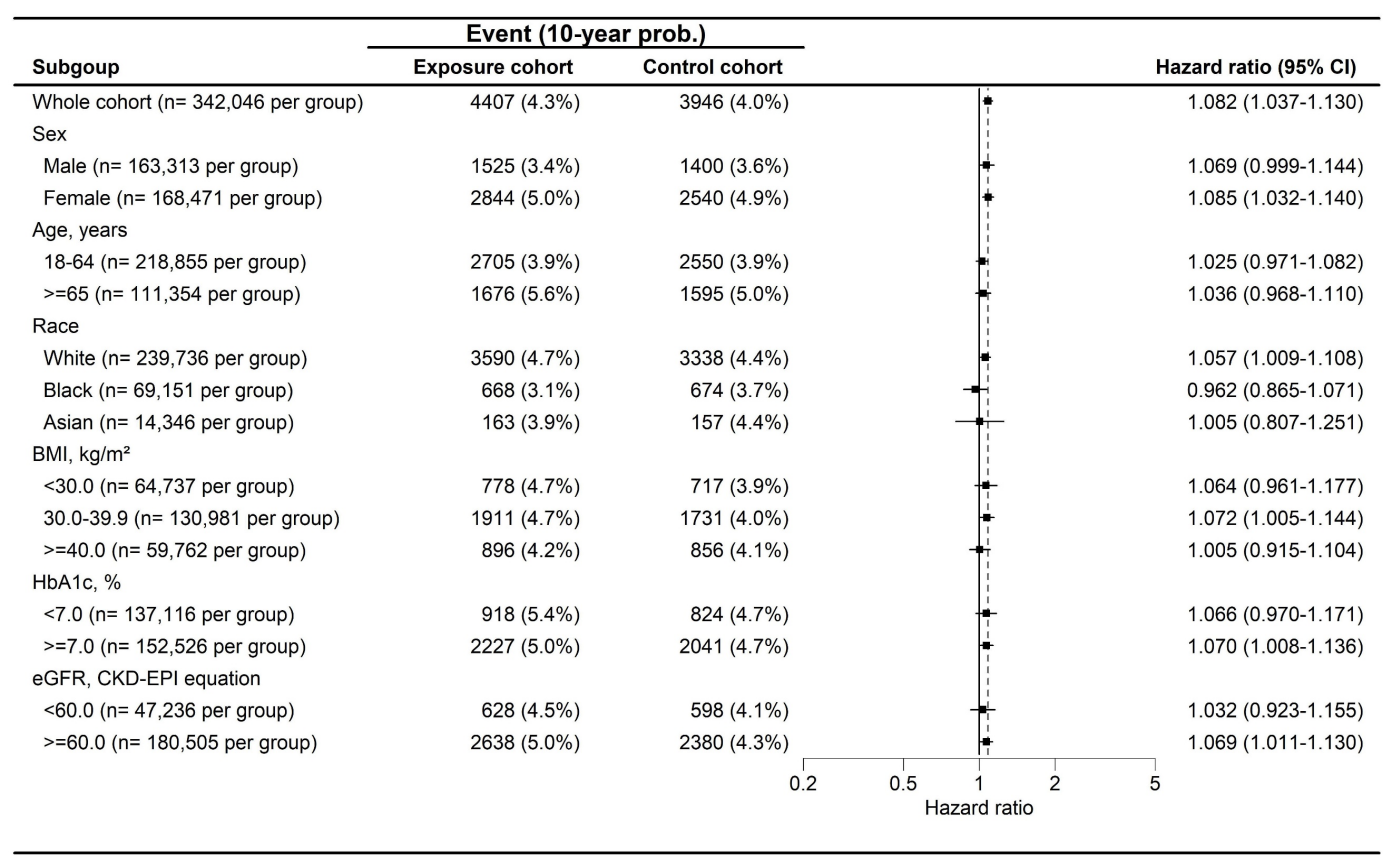
The forest plot for the cumulative probabilities of peripheral vestibular disorders in different subgroups. BMI: body mass index, CI: confidence interval, eGFR: estimated glomerular filtration rate, HbA1c: glycated hemoglobin.

**Figure 4:**
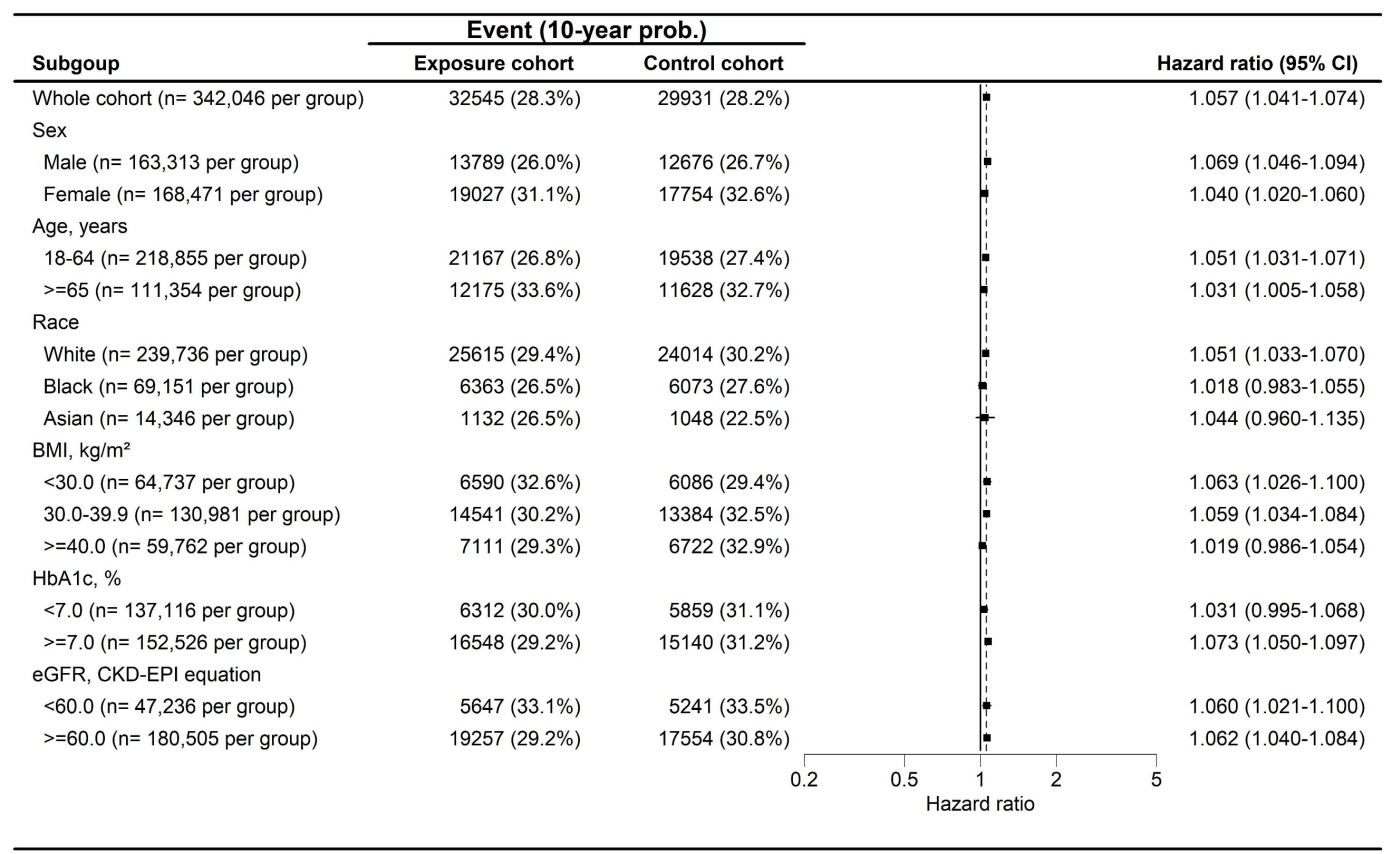
The forest plot for the cumulative probabilities of dizziness in different subgroups. BMI: body mass index, CI: confidence interval, eGFR: estimated glomerular filtration rate, HbA1c: glycated hemoglobin.

**Figure 5:**
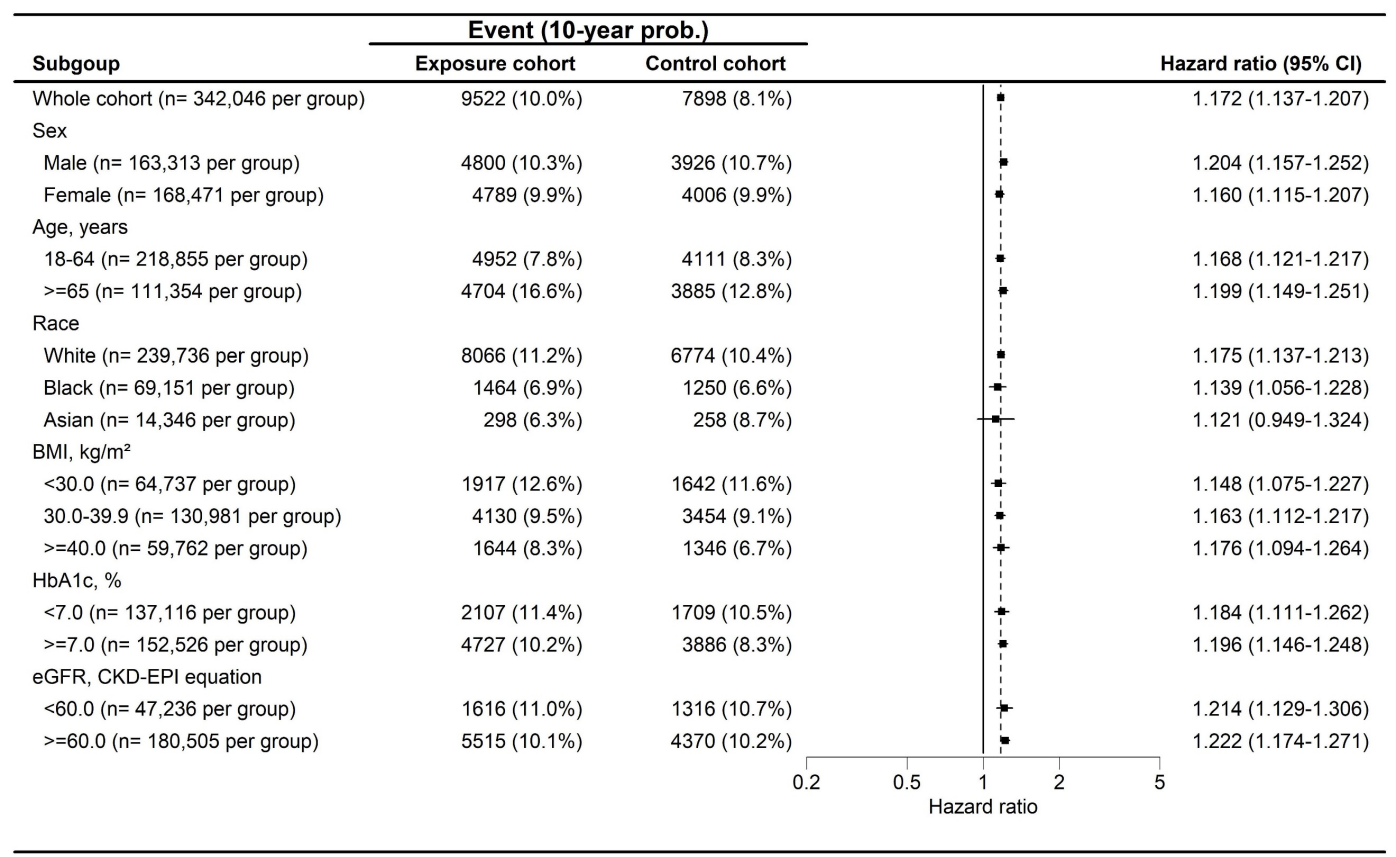
The forest plot for the cumulative probabilities of hearing impairment in different subgroups. BMI: body mass index, CI: confidence interval, eGFR: estimated glomerular filtration rate, HbA1c: glycated hemoglobin.

During a follow-up period of up to 9 years, 4,407 and 3,946 cases of peripheral vestibular disorders were identified in the GLP-1 RA and non-GLP-1 RA groups, respectively. The GLP-1 RA group exhibited a significantly higher incidence of peripheral vestibular disorders compared with the non-GLP-1 RA group [ hazard ratio (HR), 1.082; 95% confidence interval (CI), 1.037-1.130; P < 0.001] (Table 2). Dizziness occurred in 32,545 participants in the GLP-1 RA group and 29,931 participants in the non-GLP-1 RA group, with a significantly higher risk observed among GLP-1 RA users (HR, 1.057; 95% CI, 1.041-1.074; P < 0.001) (Table 2). With respect to hearing impairment, 9,522 and 7,898 events were recorded in the GLP-1 RA and non-GLP-1 RA groups, respectively. The incidence of hearing impairment was also significantly higher in the GLP-1 RA group (HR, 1.172; 95% CI, 1.137-1.207; P < 0.001) (Table 2). Kaplan-Meier analyses demonstrated that the cumulative incidences of peripheral vestibular disorders, dizziness, and hearing impairment were significantly higher in the GLP-1 RA group than in the non-LP-1 RA group (all P < 0.001) **(Figure 2A-2C)**. In subgroup analyses, GLP-1 RA users with specific characteristics, including Female, White race, body mass index (BMI) of 30-39 kg/m², HbA1c levels >7.0%, and eGFR> 60 exhibited significantly higher incidences of peripheral vestibular disorders compared with their non-GLP-1 RA counterparts, as indicated by 95% CIs exceeding unity **(Figure 3)**. Additionally, GLP-1 RA users consistently demonstrated a higher risk of dizziness across most subgroups, except among participants of Black and Asian races, BMI > 40 kg/m², and HbA1c levels < 7.0%, for whom the 95% CIs included 1 **(Figure 4)**. Similarly, GLP-1 RA use was associated with a significantly increased incidence of hearing impairment across most subgroups, except among participants of Asian race **(Figure 5)**.

## Discussion

In the present study, use of GLP-1 RAs was associated with a higher incidence of peripheral vestibular disorders, dizziness, and hearing impairment compared with non-GLP-1 RA use. In addition, the cumulative incidences of these outcomes were significantly greater among GLP-1 RA users throughout the follow-up period. The increased risks of peripheral vestibular disorders, dizziness, and hearing impairment associated with GLP-1 RA use were generally consistent across most subgroups, except among participants of Black and Asian races.

Peripheral vestibular disorders comprise a group of conditions that primarily affect the structures of the inner ear 15, 16. Benign paroxysmal positional vertigo (BPPV) is characterized by dizziness, vertigo, and spinning sensations, often accompanied by nystagmus 17. The pathophysiology of BPPV involves dislodgement of otoconia from the utricle, a process in which glycemic status may play a contributory role 17. Menière's disease is also characterized by episodic dizziness and vertigo and is thought to result from endolymphatic hydrops, electrolyte imbalance, and hyperinsulinemia, all of which may disrupt inner ear homeostasis 18, 19. Treatment options for Ménière's disease include diuretics and anti-inflammatory agents 17. Vestibular neuronitis and labyrinthitis are associated with inflammation, glycemic fluctuations, and viral infections, and anti-inflammatory therapy may be beneficial in their management 17, 18, 20.

Dizziness is a nonspecific symptom with multifactorial etiologies, including viral infection, inflammation, and fluctuations in blood glucose levels 21, 22. Hearing impairment commonly presents with muffled hearing, difficulty understanding speech, and tinnitus 23. Established risk factors for hearing loss include advanced age, noise exposure, exposure to ototoxic medications, and dysregulated glycemic status, including both hyperglycemia and hypoglycemia 24, 25. Management strategies for hearing impairment include discontinuation of ototoxic agents, use of hearing aids, and optimization of glycemic control 23. GLP-1 RAs are well-established antihyperglycemic agents with proven efficacy in patients with T2DM 26. However, GLP-1 RA therapy has been associated with adverse effects involving the gastrointestinal, biliary, and thyroid systems, which may influence electrolyte balance 12, 13, 27. Additionally, GLP-1 RAs have been reported to suppress matrix metalloproteinase-9 activity, potentially promoting a prothrombotic milieu that may impair inner ear microcirculation 14. Excessive dosing of GLP-1 RAs may also increase the risk of hypoglycemia 9. Taken together, these mechanisms provide biological plausibility for an association between GLP-1 RA use and inner ear disorders. On the other side, the liraglutide treatment, a type of GLP-1 RA, could mitigate the hearing damage and impairment induced by multiday repeated high-intensity blasts in several previous studies 28, 29, and the liraglutide also own anti-inflammatory and neuroprotective effects for neurological diseases such as stroke and Alzheimer disease 30. Consequently, additional study is warranted to evaluate the effect of GLP-1 RA on hearing system. The findings of the present study may support the hypothesis that GLP-1 RA could insult the inner ear system.

Use of GLP-1 RAs was associated with higher incidences of subsequent peripheral vestibular disorders, dizziness, and hearing impairment compared with non-GLP-1 RA use. Previous studies have reported an association between hypoglycemic states and the occurrence of peripheral vestibular disorders 31. In addition, prior research has demonstrated that the magnitude of glycemic reduction achieved with GLP-1 RAs is greater than that observed with DPP-4i 26. However, to date, no studies have specifically evaluated the potential association between GLP-1 RA use and the subsequent development of peripheral vestibular disorders or hearing impairment. To our knowledge, the findings of the present study provide preliminary evidence suggesting a possible association between GLP-1 RA administration and the subsequent development of peripheral vestibular disorders and hearing impairment. Importantly, participants with diagnoses of peripheral vestibular disorders, dizziness, or hearing impairment before the index date or during the washout period were excluded, thereby strengthening the temporal relationship between exposure and outcomes. Furthermore, multiple established risk factors for peripheral vestibular disorders and hearing impairment, including age, cardiovascular disease, use of ototoxic medications, and history of middle ear surgery, were accounted for and balanced in the Cox proportional hazards regression models 18, 23. Therefore, GLP-1 RA use may be independently associated with an increased risk of subsequent peripheral vestibular disorders and hearing impairment. Cumulative incidence analyses further demonstrated consistently higher risks of peripheral vestibular disorders, dizziness, and hearing impairment in the GLP-1 RA group. These findings suggest that long-term users of GLP-1 RAs may warrant closer clinical monitoring for potential auditory and vestibular complications compared with short-term users.

Regarding the sensitivity analyses, GLP-1 RA users with various clinical characteristics generally demonstrated higher incidences of peripheral vestibular disorders, dizziness, and hearing impairment compared with non-GLP-1 RA users, except among participants of Black and Asian races. Previous studies have identified advanced age as a major risk factor for the development of both peripheral vestibular disorders and hearing impairment 16, 23. In addition, female sex and metabolic conditions, including obesity, have been associated with an increased risk of BPPV 16, 32. Poor glycemic control has also been linked to the development of hearing impairment in prior literature 23. Accordingly, it is plausible that individuals with these characteristics who received GLP-1 RA therapy exhibited higher incidences of peripheral vestibular disorders, dizziness, and hearing impairment than their counterparts who did not receive GLP-1 RA treatment. In contrast, GLP-1 RA users of Black and Asian races did not demonstrate significantly higher incidences of peripheral vestibular disorders, dizziness, or hearing impairment compared with nonusers of the same racial groups. Limited evidence exists to explain this finding. One possible explanation is the relatively small sample size of these subpopulations, as only approximately 24% of participants were of Black or Asian race. A similar limitation may apply to participants with a BMI greater than 40 kg/m², who comprised only 17% of the study population. The lack of statistical significance in these subgroups may therefore be attributable to insufficient statistical power, and differences between GLP-1 RA and non-GLP-1 RA groups may emerge with larger sample sizes. Further studies are warranted to confirm this hypothesis. From an epidemiological perspective, T2DM is a highly prevalent disease worldwide, affecting more than 500 million individuals, with a reported prevalence of approximately 14% among elderly populations in China 2, 33. GLP-1 RAs are commonly prescribed in patients with T2DM, and at least seven GLP-1 RA agents are currently available globally 9. Similarly, dizziness is one of the most common clinical complaints, with a lifetime prevalence ranging from 15% to 35% 22. Hearing impairment affects a substantial proportion of the population, with an estimated prevalence of 67% among individuals aged 70 years or older in the United States 24, 34. Given the large and overlapping populations of GLP-1 RA users and individuals affected by these inner ear disorders, any potential association between GLP-1 RA use and auditory or vestibular outcomes warrants careful investigation and clinical attention.

Several limitations of the present study should be acknowledged. First, the TriNetX database captures structured data such as demographic information, diagnostic codes, laboratory results, procedures, and medication records, but does not include detailed clinical narratives. Consequently, several important variables were unavailable, including the duration and clinical course of T2DM, actual dosages and cumulative exposure to antidiabetic medications, medication adherence, detailed symptom profiles of peripheral vestibular disorders, treatment strategies and prognoses for vestibular disorders and dizziness, pure-tone audiometry results, laterality and management of hearing impairment, and detailed information on other systemic conditions. Second, the retrospective design of this study may have introduced residual confounding and reduced cohort homogeneity, which could influence the results. Although propensity score matching achieved good balance in baseline characteristics between the two groups, differences related to unmeasured confounders may still exist which include the impacts of lifestyle, noise exposure, family history, medication adherence. Third, while exposure to SGLT2 inhibitors and DPP-4 inhibitors was excluded in the GLP-1 RA group, the potential effects of other antidiabetic medications could not be fully eliminated and may have influenced outcome assessment. Some participants may have received self-paid GLP-1 RA treatments (e.g., tirzepatide) that were not captured in the TriNetX system and were therefore misclassified as non-GLP-1 RA users, potentially leading to exposure misclassification and bias toward the null. The possible mechanisms for the correlation between GLP-1 RA treatments to peripheral vestibular disorders and hearing impairment were not confirmed by laboratory evaluation due to the database nature. Accordingly, the negative effect of GLP-1 RA treatments on the peripheral vestibular disorders and hearing impairment might be an observed finding rather than a causal relationship. Finally, the major population of TriNetX database is the white population and we excluded distinctions for some racial groups like the Hispanic ethnicity, thus the generalizability of our results may be low especially for the non-white populations considering the existing differences in disease prevalence and healthcare access across groups/ethnicities.

In conclusion, GLP-1 RA use may be presented with higher incidences of peripheral vestibular disorders and hearing impairment compared with non-GLP-1 RA use after adjustment for multiple confounders in white population. Furthermore, the risk of developing these inner ear disorders was observed in those with longer durations of GLP-1 RA exposure. These findings suggest that patients with T2DM receiving long-term GLP-1 RA therapy may benefit from routine ontological evaluation for early detection of vestibular and auditory disorders. Further large-scale prospective studies are warranted to elucidate the relationship between GLP-1 RA use and peripheral vestibular disorders and hearing impairment.

## Figures and Tables

**Table 1:** Baseline characteristics among GLP-1 RA cohort and Non-GLP-1 RA cohort before and after propensity score matching.

	Before matching			After propensity score matching		
	GLP-1 RA cohort	Non-GLP-1 RA cohort	SMD	GLP-1 RA cohort	Non-GLP-1 RA cohort	SMD
N	481014	778282		342046	342046	
Age at Index	55.7 ± 13.0	62.2 ± 12.3	0.5102	58.5 ± 12.0	58.5 ± 12.8	0.0028
Sex						
Female	277928 (57.8%)	333582 (42.9%)	0.3017	175910 (51.4%)	175174 (51.2%)	0.0043
Male	202522 (42.1%)	443804 (57.0%)	0.3018	165633 (48.4%)	166492 (48.7%)	0.0050
Race						
White	315164 (65.5%)	428266 (55.0%)	0.2157	217101 (63.5%)	218795 (64.0%)	0.0103
Black or African American	98176 (20.4%)	127104 (16.3%)	0.1055	65994 (19.3%)	66016 (19.3%)	0.0002
Asian	15389 (3.2%)	64028 (8.2%)	0.2179	13927 (4.1%)	13646 (4.0%)	0.0042
Hazards related to socioeconomic and psychosocial circumstances	6829 (1.4%)	11443 (1.5%)	0.0042	4136 (1.2%)	4858 (1.4%)	0.0185
Medical encounter						
Preventive Medicine Services	69273 (14.4%)	47573 (6.1%)	0.2758	32265 (9.4%)	33231 (9.7%)	0.0096
Inpatient Encounter	47259 (9.8%)	171056 (22.0%)	0.3370	41273 (12.1%)	42210 (12.3%)	0.0084
Emergency	62066 (12.9%)	125988 (16.2%)	0.0933	45625 (13.3%)	47038 (13.8%)	0.0121
Lifestyle						
Nicotine dependence	31526 (6.6%)	57335 (7.4%)	0.0319	22947 (6.7%)	23318 (6.8%)	0.0043
Alcohol related disorders	5223 (1.1%)	12972 (1.7%)	0.0499	4118 (1.2%)	4176 (1.2%)	0.0015
Comorbidities						
Type 2 diabetes mellitus with ophthalmic complications	14682 (3.1%)	24229 (3.1%)	0.0035	12254 (3.6%)	9719 (2.8%)	0.0420
Hypoglycemia	2032 (0.4%)	4593 (0.6%)	0.0236	1521 (0.4%)	1435 (0.4%)	0.0038
Dyslipidemia	240175 (49.9%)	406873 (52.3%)	0.0470	172831 (50.5%)	173029 (50.6%)	0.0012
Hypertensive diseases	263033 (54.7%)	460943 (59.2%)	0.0918	189469 (55.4%)	190379 (55.7%)	0.0054
Diseases of liver	32613 (6.8%)	47053 (6.0%)	0.0300	20895 (6.1%)	20821 (6.1%)	0.0009
Chronic kidney disease	31429 (6.5%)	96248 (12.4%)	0.2004	27732 (8.1%)	28970 (8.5%)	0.0131
Ischemic heart diseases	46196 (9.6%)	157421 (20.2%)	0.3016	41163 (12.0%)	42552 (12.4%)	0.0124
Heart failure	20040 (4.2%)	100584 (12.9%)	0.3172	19329 (5.7%)	21220 (6.2%)	0.0234
Cerebrovascular diseases	14751 (3.1%)	52591 (6.8%)	0.1714	13084 (3.8%)	13582 (4.0%)	0.0075
Transient cerebral ischemic attacks	2714 (0.6%)	7941 (1.0%)	0.0515	2290 (0.7%)	2323 (0.7%)	0.0012
Chronic lower respiratory diseases	58791 (12.2%)	90193 (11.6%)	0.0196	38269 (11.2%)	38997 (11.4%)	0.0067
Mood disorders	72315 (15.0%)	74444 (9.6%)	0.1671	40650 (11.9%)	41380 (12.1%)	0.0066
Anxiety disorders	73790 (15.3%)	75805 (9.7%)	0.1697	40432 (11.8%)	41313 (12.1%)	0.0079
Schizophrenia	1677 (0.3%)	4337 (0.6%)	0.0311	1382 (0.4%)	1444 (0.4%)	0.0028
Delirium	445 (0.1%)	2770 (0.4%)	0.0557	430 (0.1%)	515 (0.2%)	0.0067
Rheumatoid arthritis	5183 (1.1%)	7346 (0.9%)	0.0134	3390 (1.0%)	3435 (1.0%)	0.0013
Superficial injury of the head	1579 (0.3%)	4655 (0.6%)	0.0397	1296 (0.4%)	1286 (0.4%)	0.0005
Open wound of head	1210 (0.3%)	3437 (0.4%)	0.0323	993 (0.3%)	1023 (0.3%)	0.0016
Migraine	14343 (3.0%)	9671 (1.2%)	0.1212	6468 (1.9%)	6668 (1.9%)	0.0043
Otitis media	3436 (0.7%)	2777 (0.4%)	0.0490	1765 (0.5%)	1657 (0.5%)	0.0045
Medication						
SGLT2 inhibitors	0 (0.0%)	415090 (53.3%)	1.5119	0 (0.0%)	204971 (59.9%)	1.7293
DPP-4 inhibitors	0 (0.0%)	391931 (50.4%)	1.4244	0 (0.0%)	149252 (43.6%)	1.2443
Metformin	223352 (46.4%)	436482 (56.1%)	0.1940	174195 (50.9%)	172118 (50.3%)	0.0121
Sulfonylureas	65403 (13.6%)	179488 (23.1%)	0.2465	57770 (16.9%)	58056 (17.0%)	0.0022
Pioglitazone	12959 (2.7%)	34835 (4.5%)	0.0959	11340 (3.3%)	11472 (3.4%)	0.0021
Insulin	134876 (28.0%)	245742 (31.6%)	0.0773	104011 (30.4%)	105278 (30.8%)	0.0080
Statins	216118 (44.9%)	451578 (58.0%)	0.2642	172767 (50.5%)	172319 (50.4%)	0.0026
Beta-blockers	110194 (22.9%)	282110 (36.2%)	0.2954	91119 (26.6%)	92430 (27.0%)	0.0087
ACE inhibitors	111656 (23.2%)	202435 (26.0%)	0.0650	86574 (25.3%)	87219 (25.5%)	0.0043
ARBs	97574 (20.3%)	222786 (28.6%)	0.1950	76739 (22.4%)	76316 (22.3%)	0.0030
Calcium channel blockers	89549 (18.6%)	213176 (27.4%)	0.2096	71842 (21.0%)	72107 (21.1%)	0.0019
Diuretics	135821 (28.2%)	262430 (33.7%)	0.1188	101320 (29.6%)	102549 (30.0%)	0.0079
Aspirin	60921 (12.7%)	196018 (25.2%)	0.3238	53255 (15.6%)	59676 (17.4%)	0.0506
NSAIDs	111522 (23.2%)	152222 (19.6%)	0.0885	76639 (22.4%)	70635 (20.7%)	0.0427
Systemic corticosteroids	125759 (26.1%)	186598 (24.0%)	0.0501	83151 (24.3%)	84089 (24.6%)	0.0064
Orlistat	215 (0.0%)	71 (0.0%)	0.0217	112 (0.0%)	40 (0.0%)	0.0141
Phentermine	8656 (1.8%)	1833 (0.2%)	0.1563	4378 (1.3%)	1293 (0.4%)	0.0996
Topiramate	10261 (2.1%)	6201 (0.8%)	0.1114	5714 (1.7%)	3788 (1.1%)	0.0481
Naltrexone	2717 (0.6%)	1090 (0.1%)	0.0717	1498 (0.4%)	623 (0.2%)	0.0460
Bupropion	23991 (5.0%)	18241 (2.3%)	0.1410	14624 (4.3%)	10281 (3.0%)	0.0678
Aminoglycosides	9828 (2.0%)	24275 (3.1%)	0.0679	7546 (2.2%)	7601 (2.2%)	0.0011
Macrolides	31322 (6.5%)	45387 (5.8%)	0.0283	20685 (6.0%)	20875 (6.1%)	0.0023
Loop diuretics	42581 (8.9%)	130752 (16.8%)	0.2394	36580 (10.7%)	38170 (11.2%)	0.0149
Cisplatin	74 (0.0%)	681 (0.1%)	0.0318	62 (0.0%)	143 (0.0%)	0.0137
Carboplatin	172 (0.0%)	814 (0.1%)	0.0260	134 (0.0%)	276 (0.1%)	0.0170
Vancomycin	12624 (2.6%)	37983 (4.9%)	0.1189	10838 (3.2%)	11316 (3.3%)	0.0079
Surgery procedures						
Sleeve gastrectomy	31 (0.0%)	181 (0.0%)	0.0138	19 (0.0%)	38 (0.0%)	0.0061
Roux-en-Y gastric bypass	94 (0.0%)	58 (0.0%)	0.0104	42 (0.0%)	48 (0.0%)	0.0015
Surgical Procedures on the Middle Ear	155 (0.0%)	166 (0.0%)	0.0067	99 (0.0%)	79 (0.0%)	0.0036
Healthcare utilization						
Preventive medicine services	69273 (14.4%)	47573 (6.1%)	0.2758	32265 (9.4%)	33231 (9.7%)	0.0096
Emergency department visits	62066 (12.9%)	125988 (16.2%)	0.0933	45625 (13.3%)	47038 (13.8%)	0.0121
Inpatient encounters	47259 (9.8%)	171056 (22.0%)	0.3370	41273 (12.1%)	42210 (12.3%)	0.0084
Lab data						
BMI	37.6 ± 8.3	32.3 ± 7.5	0.6733	35.6 ± 7.8	35.0 ± 7.6	0.0825
eGFR	83.7 ± 26.6	77.8 ± 29.2	0.2124	81.2 ± 26.9	81.6 ± 28.7	0.0163
HbA1c	8.0 ± 2.1	8.2 ± 2.0	0.0854	8.2 ± 2.1	8.3 ± 2.1	0.0259
LDLc	96.9 ± 38.6	90.3 ± 39.3	0.1703	93.3 ± 39.1	93.5 ± 39.4	0.0059

BMI: body mass index, DPP-4i: dipeptidyl peptidase-4 inhibitor, eGFR: estimated glomerular filtration rate, GLP-1 RA: glucagon-like peptide-1 receptor agonist, HbA1c: glycated hemoglobin, LDL: low-density lipoprotein, N: number, NSAIDs: nonsteroidal anti-inflammatory drugs, SGLT2i: sodium-glucose cotransporter 2 inhibitors, SMD: standardized mean difference * denotes significant difference between groups

**Table 2:** Main outcomes between the two groups.

Study event	N of event	Cumulative probability of study event since index date			Hazard ratio (95% CI)	*P* value
		1-year	5-year	10-years		
Peripheral vestibular disorders						
GLP-1 RA cohort	4,407	0.29%	1.87%	4.26%	1.082 (1.037-1.130)	<0.001*
Non-GLP-1 RA cohort	3,946	0.28%	1.79%	3.97%	Reference	
Dizziness symptoms						
GLP-1 RA cohort	32,545	2.51%	13.89%	28.31%	1.057 (1.041-1.074)	<0.001*
Non-GLP-1 RA cohort	29,931	2.40%	13.11%	28.23%	Reference	
Hearing impairment						
GLP-1 RA cohort	9,522	0.65%	4.08%	10.00%	1.172 (1.137-1.207)	<0.001*
Non-GLP-1 RA cohort	7,898	0.54%	3.46%	8.05%	Reference	

CI: confidence interval, GLP-1 RA: glucagon-like peptide-1 receptor agonist, N: number * denotes significant difference between groups

## References

[1] Stumvoll M, Goldstein BJ, van Haeften TW (2005). Type 2 diabetes: principles of pathogenesis and therapy. Lancet.

[2] Ahmad E, Lim S, Lamptey R, Webb DR, Davies MJ (2022). Type 2 diabetes. Lancet.

[3] Chatterjee S, Khunti K, Davies MJ (2017). Type 2 diabetes. Lancet.

[4] Thipsawat S (2021). Early detection of diabetic nephropathy in patient with type 2 diabetes mellitus: A review of the literature. Diab Vasc Dis Res.

[5] Tan SY, Mei Wong JL, Sim YJ, Wong SS, Mohamed Elhassan SA, Tan SH (2019). Type 1 and 2 diabetes mellitus: A review on current treatment approach and gene therapy as potential intervention. Diabetes Metab Syndr.

[6] Artasensi A, Pedretti A, Vistoli G, Fumagalli L (2020). Type 2 Diabetes Mellitus: A Review of Multi-Target Drugs. Molecules.

[7] Bidulka P, Lugo-Palacios DG, Carroll O, O'Neill S, Adler AI, Basu A (2024). Comparative effectiveness of second line oral antidiabetic treatments among people with type 2 diabetes mellitus: emulation of a target trial using routinely collected health data. Bmj.

[8] Pratley RE, Heller SR, Miller MA (2014). Treatment of type 2 diabetes mellitus in the older adult: a review. Endocr Pract.

[9] Trujillo JM, Nuffer W, Smith BA (2021). GLP-1 receptor agonists: an updated review of head-to-head clinical studies. Ther Adv Endocrinol Metab.

[10] Karagiannis T, Avgerinos I, Liakos A, Del Prato S, Matthews DR, Tsapas A (2022). Management of type 2 diabetes with the dual GIP/GLP-1 receptor agonist tirzepatide: a systematic review and meta-analysis. Diabetologia.

[11] Moiz A, Filion KB, Tsoukas MA, Yu OH, Peters TM, Eisenberg MJ (2025). Mechanisms of GLP-1 Receptor Agonist-Induced Weight Loss: A Review of Central and Peripheral Pathways in Appetite and Energy Regulation. Am J Med.

[12] Jalleh RJ, Plummer MP, Marathe CS, Umapathysivam MM, Quast DR, Rayner CK (2024). Clinical Consequences of Delayed Gastric Emptying With GLP-1 Receptor Agonists and Tirzepatide. J Clin Endocrinol Metab.

[13] Silverii GA, Marinelli C, Bettarini C, Del Vescovo GG, Monami M, Mannucci E (2025). GLP-1 receptor agonists and the risk for cancer: A meta-analysis of randomized controlled trials. Diabetes Obes Metab.

[14] Chen J-J, Hsu C-W, Hung C-M, Liang C-S, Su K-P, Carvalho AF (2025). Risk of Hearing Loss in Patients Treated with Exendin-4 Derivatives: A Network Meta-Analysis of Glucagon-like Peptide-1 Receptor Agonists and Sodium-Glucose Cotransporter 2 Inhibitors. Pharmaceuticals.

[15] Dunlap PM, Holmberg JM, Whitney SL (2019). Vestibular rehabilitation: advances in peripheral and central vestibular disorders. Curr Opin Neurol.

[16] Yang TH, Xirasagar S, Cheng YF, Wu CS, Kuo NW, Lin HC (2021). Peripheral Vestibular Disorders: Nationwide Evidence From Taiwan. Laryngoscope.

[17] Strupp M, Dlugaiczyk J, Ertl-Wagner BB, Rujescu D, Westhofen M, Dieterich M (2020). Vestibular Disorders. Dtsch Arztebl Int.

[18] Strupp M, Brandt T (2013). Peripheral vestibular disorders. Curr Opin Neurol.

[19] Spencer JT Jr (1981). Hyperlipoproteinemia, hyperinsulinism, and Meniere's disease. South Med J.

[20] Yang T-H, Chen C-H, Cheng Y-F, Lin H-C, Chen C-S (2024). Association of Peripheral Vestibular Disorder with Diabetes: A Population-Based Study. Journal of Personalized Medicine.

[21] Staab JP (2023). Persistent Postural-Perceptual Dizziness: Review and Update on Key Mechanisms of the Most Common Functional Neuro-otologic Disorder. Neurol Clin.

[22] De Vestel C, Vereeck L, Reid SA, Van Rompaey V, Lemmens J, De Hertogh W (2022). Systematic review and meta-analysis of the therapeutic management of patients with cervicogenic dizziness. J Man Manip Ther.

[23] Cunningham LL, Tucci DL (2017). Hearing Loss in Adults. N Engl J Med.

[24] Nieman CL, Oh ES (2020). Hearing Loss. Ann Intern Med.

[25] Sohmer H (1997). Pathophysiological mechanisms of hearing loss. J Basic Clin Physiol Pharmacol.

[26] Gilbert MP, Pratley RE (2020). GLP-1 Analogs and DPP-4 Inhibitors in Type 2 Diabetes Therapy: Review of Head-to-Head Clinical Trials. Front Endocrinol (Lausanne).

[27] He L, Wang J, Ping F, Yang N, Huang J, Li Y (2022). Association of Glucagon-Like Peptide-1 Receptor Agonist Use With Risk of Gallbladder and Biliary Diseases: A Systematic Review and Meta-analysis of Randomized Clinical Trials. JAMA Intern Med.

[28] Jiang S, Cai Q, Bien AG, Gan RZ, Jiang Y (2025). Effects of Liraglutide on Mitigation of Hearing Loss After Repeated Blast Exposures: A Summary of Studies in Animal Model of Chinchilla. Mil Med.

[29] Jiang S, Cai Q, Jiang Y, Gan RZ (2025). Effect of liraglutide treatment on mitigation of hearing damage induced by multiday repeated high-intensity blasts. J Acoust Soc Am.

[30] Candeias EM, Sebastião IC, Cardoso SM, Correia SC, Carvalho CI, Plácido AI (2015). Gut-brain connection: The neuroprotective effects of the anti-diabetic drug liraglutide. World J Diabetes.

[31] Serra AP, Lopes Kde C, Dorigueto RS, Ganança FF (2009). Blood glucose and insulin levels in patients with peripheral vestibular disease. Braz J Otorhinolaryngol.

[32] Demircan SK, Öner F (2025). The Association of Sarcopenia and Body Composition With Benign Positional Paroxysmal Vertigo in Older Adults. Laryngoscope.

[33] Yang L, Shao J, Bian Y, Wu H, Shi L, Zeng L (2016). Prevalence of type 2 diabetes mellitus among inland residents in China (2000-2014): A meta-analysis. J Diabetes Investig.

[34] Sheffield AM, Smith RJH (2019). The Epidemiology of Deafness. Cold Spring Harb Perspect Med.

